# Gene silencing by RNA interference in the ectoparasitic mite, *Psoroptes ovis*

**DOI:** 10.1186/s13567-018-0608-9

**Published:** 2018-11-01

**Authors:** Edward J. Marr, Harry W. Wright, Neil D. Sargison, Alasdair J. Nisbet, Stewart T. G. Burgess

**Affiliations:** 1 Moredun Research Institute, Pentlands Science Park, Bush Loan, Penicuik, Edinburgh, EH26 0PZ Scotland, UK; 2 Royal (Dick) School of Veterinary Studies, University of Edinburgh, Easter Bush, Roslin, Midlothian, EH25 9RG Scotland, UK; 3 Institute of Immunology and Infection Research, The King’s Buildings, Ashworth Laboratories, Charlotte Auerbach Road, Edinburgh, EH9 3FL Scotland, UK

## Abstract

The presence of components of the RNA interference (RNAi) pathway in *Psoroptes ovis*, an ectoparasitic mite responsible for psoroptic mange, was investigated through interrogation of the *P. ovis* genome. Homologues of transcripts representing critical elements for achieving effective RNAi in the mite, *Tetranychus urticae* and the model organisms *Caenorhabditis elegans* and *Drosophila melanogaster* were identified and, following the development of a non-invasive immersion method of double stranded RNA delivery, gene silencing by RNAi was successfully demonstrated in *P. ovis.* Significant reductions in transcript levels were achieved for three target genes which encode the Group 2 allergen (Pso o 2), mu-class glutathione *S*-transferase (*Po*GST-mu1) and beta-tubulin (*Po*βtub). This is the first demonstration of RNAi in *P. ovis* and provides a mechanism for mining transcriptomic and genomic datasets for novel control targets against this economically important ectoparasite.

## Introduction

Psoroptic mange, caused by infestation with the ectoparasitic mite *Psoroptes ovis*, Hering, 1838, is a disease of significant welfare and economic importance with a global presence. Affecting production animals such as sheep, cattle, bison, water buffalo, camelids and rabbits and wildlife including giraffe and bighorn sheep [1–5], *Psoroptes* sp. are responsible for diseases that are typically highly contagious and cause intense inflammatory responses accompanied by extreme pruritis, leading to serious welfare concerns [6]. *Psoroptes ovis* infestation in sheep leads to the disease known as sheep scab and results in economic losses as a result of, for example, costs accrued through prevention and treatment, damage to wool and leather quality, rejection of carcasses at slaughter and fatalities due to hypothermia and epileptiform convulsions [7–14]. Although dipping of sheep in organophosphate acaricides largely remains effective, it is potentially hazardous to the users, traumatic for sheep and can give rise to serious environmental concerns [15, 16]. Resistance to some of the compounds used to treat and control *P. ovis* has been recorded, some of which are also relied upon for the control of gastrointestinal nematodes, thereby increasing the risk of selecting for further resistance [7]. More recently, resistance to the macrocyclic lactone compound, moxidectin, has been confirmed in populations of *P. ovis* in England and Wales, placing further pressure on the existing methods of control and highlighting the need for novel means of control, such as vaccination [17].

The first draft genome for *P. ovis* was recently published [18] providing a valuable resource for molecular studies into this ectoparasite. This new genome resource can be exploited for the identification of novel vaccine and diagnostic candidates and also for molecular genetic studies into the development of acaricide resistance. However, these studies will be further enhanced by an effective means of investigating gene function in *P. ovis*. We therefore interrogated the *P. ovis* genome to identify components of the RNAi machinery and then, based on the outcomes of that process and previous studies in other closely related astigmatid mites [19, 20], we sought to develop a non-invasive means of RNAi in *P. ovis*. These new tools provide a crucial resource for the identification and validation of novel means of control for this economically important ectoparasite.

## Materials and methods

### Identification of *P. ovis* RNAi pathway genes

The *P. ovis* genome (DDBJ/ENA/GenBank accession PQWQ00000000) is predicted to encode 12 041 protein-coding genes, of which annotation has been achieved at the gene ontology (GO) level for 7957 genes, with functional annotation of 5217 genes [18]. The genome was interrogated for homologues of known RNAi pathway components identified from the two-spotted spider mite, *Tetranychus urticae* and the model organisms *Caenorhabditis elegans* and *Drosophila melanogaster*. Predicted coding sequences of the major proteins involved in RNAi biosynthesis and processing in these other species were obtained from previously-published analyses [21] and were used to search the predicted coding sequences from the *P. ovis* genome (as a reference database). A tBLASTx analysis was performed using BLAST^®^ [22] with each sequence (target and reference) being translated into each of the 6 possible reading frames and a significance threshold of < 1e−10^−4^ applied. Phylogenetic clustering of *P. ovis* argonaute proteins was performed in CLC Genomics Workbench 11.0 (Qiagen Ltd, UK).

### Harvesting of *P. ovis* mites

Mixed populations (mixture of all life stages) of *P. ovis* were harvested from skin sections taken from experimentally-infested donor sheep, under the legislation of a UK Home Office Project License (reference PPL 60/4238) in accordance with the Animals (Scientific Procedures) Act of 1986. Ethical approval for the maintenance of this model was obtained from the Moredun Research Institute local ethics committee [E03/17] and animals were monitored daily in accordance with UK Home Office guidelines.

### Validation of non-invasive RNAi methodology in *P. ovis* mites

A non-invasive immersion technique for RNAi developed in *Varroa destructor* [23] was adapted for *P. ovis* using modifications [20]. Briefly, mites, or their eggs, were immersed in the detached cap of a 1.5 mL micro-centrifuge tube in 15 µL of a solution containing either a fluorophore-labelled AllStars™ Negative Control siRNA (Qiagen, UK) (“fluoro-siRNA”) in 0.9% w/v NaCl solution, final fluoro-siRNA concentration 0.05 µg/µL, or 15 µL 0.9% w/v NaCl solution only. The mites were incubated at 4 °C overnight then washed three times in molecular biology grade water (Sigma, UK) before photographic images were captured through an Axiovert 25 CFL inverted fluorescent microscope (Zeiss, Germany) with 10× Achrostigmat magnification lens (Zeiss, Germany) using a D90 AF-5 DX NIKKOR digital camera with 18–105 mm f/3.5–5.6 G ED VR lens kit (Nikon, Japan).

### Preparation of gene-specific dsRNA

Double stranded RNA (dsRNA) encoding 300 bp of a *P. ovis* major allergen (Pso o 2) (Gene ID: psovi17g08010), glutathione-*S*-transferase mu class 1 (*Po*GST-mu1) (Gene ID: psovi284g01800), 500 bp beta tubulin (*Po*βtub) (Gene ID: psovi22g01550) or 319 bp *Escherichia coli* strain K-12 sub-strain MG1655 lacZ (NC_000913.3) was produced using the dsRNA production vector pL4440 from the Fire Lab *C. elegans* Vector Kit 1999 (Addgene plasmid #1654). pL4440 vector containing the target gene sequence was produced by ligating the target gene into pL4440 using *Sac*1 and *Sma*1 restriction sites (Roche, UK). Primer sequences are available from the authors on request. Double stranded RNA (dsRNA) was synthesised from linearised plasmid using the T7 RiboMAX™ Express RNAi System (Promega, UK) according to the manufacturer’s protocol. The quality of dsRNA was assessed using gel electrophoresis and the concentration determined using an ND-1000 Nanodrop spectrophotometer (Thermo Scientific, UK).

### Gene silencing by non-invasive RNAi in *P. ovis* mites

Twenty adult male *P. ovis* mites were immersed in 15 µL dsRNA encoding either Pso o 2, *Po*GST-mu1, *Po*βtub or lacZ, diluted in 0.9% w/v NaCl solution to a final concentration of 2.5 µg/µL in the detached cap of a 1.5 mL micro-centrifuge tube (Axygen, USA). Five replicates per treatment group were performed for each experiment. The mites were then either: (i) incubated at 4 °C for 24 h and then processed for RNA extraction or, (ii) incubated at 4 °C for 24 h, dried using filter paper (Whatman, UK) and then subjected to a second incubation step (without immersion in dsRNA) in an incubator for 48 h at 25 °C, 75% relative humidity. Following both protocols, RNA was extracted and the quality assessed as previously described [20].

### cDNA synthesis for qPCR

First strand cDNA synthesis was performed using Superscript II (Invitrogen, UK) and oligo(dT)_23_ anchored primers (Sigma-Aldrich, UK) using *P. ovis* total RNA extracted using ZR Tissue & Insect Microprep Kit (Zymo Research, USA). *Psoroptes ovis* total RNA (10 µL), 1 µL dNTP mix (10 mM each) and 1 µL (0.7 µM) oligo(dT)_23_ anchored primers (Sigma-Aldrich, UK) were incubated at 65 °C for 5 min then transferred to ice. 5× First Strand Synthesis Buffer (4 µL), 2 µL DTT (1 M) and 1 µL (40 units) RNaseOUT™ Recombinant Ribonuclease Inhibitor (Invitrogen, UK) were added, gently mixed and incubated at 42 °C for 2 min before incubation on ice and addition of 1 µL (200 Units) SuperScript^®^ II Reverse Transcriptase (Invitrogen, UK). The samples were then incubated at 42 °C for 50 min, 70 °C for 15 min and resulting cDNA was stored at −20 °C until use.

### Quantitative PCR (qPCR) assessment of gene silencing in *P. ovis*

Gene-specific primers were designed for the amplification of the transcripts encoding Pso o 2, *Po*GST-mu1, *Po*βtub and *E*. *coli* lacZ using Primer3 and were sited outside of the gene region used for the construction of the dsRNA [24]. Primers for *Po*βactin were designed as described in Burgess et al. [25]. qPCR reactions were performed in triplicate on cDNA samples (diluted 1:3 with molecular grade water (Sigma, UK)) using an ABI Prism 7500 real-time thermal cycler (Applied Biosystems, UK); qPCR cycling times were 50 °C for 2 min then 95 °C for 5 min, followed by 40 cycles of 95 °C for 30 s, 57 °C for 40 s, and 72 °C for 40 s. Melt curve analysis cycle times were 95 °C for 15 s, 60 °C for 60 s and 95 °C for 15 s. A single 25 μL PCR reaction consisted of 1 μL cDNA, 12.5 μL SYBR^®^ GreenER™ qPCR SuperMix for ABI PRISM^®^ (Life Technologies, UK), 0.5 μL of each primer (2 μM) and 10.5 μL of molecular biology grade water (Sigma, UK). Standard curves (10^8^–10^2^ copies/μL) were prepared from 10^9^ copies/μL stock of each plasmid DNA and amplified in triplicate. Correlation co-efficients of the standard curves were used to calculate PCR efficiencies, which averaged > 90%. The average number of copies/μL of cDNA was calculated, and the results were normalised to the reference gene, *Po*βactin. Melt curve analysis showed single amplicons in all cases and these were validated by Sanger sequencing.

### Statistical analysis

Statistical analysis was performed using GraphPad Prism 6 (GraphPad Software, UK). A Student’s *t*-test was used to determine the statistical difference between the means of the two treatment groups in each experiment. Data were assessed for normal distribution using Minitab^®^ version 17 (Minitab Ltd, UK) and log transformations of raw data were applied as appropriate, *p*-values < 0.05 were considered significant.

## Results

### Identification of RNAi pathway genes in the *P. ovis* genome

**Figure 1:**
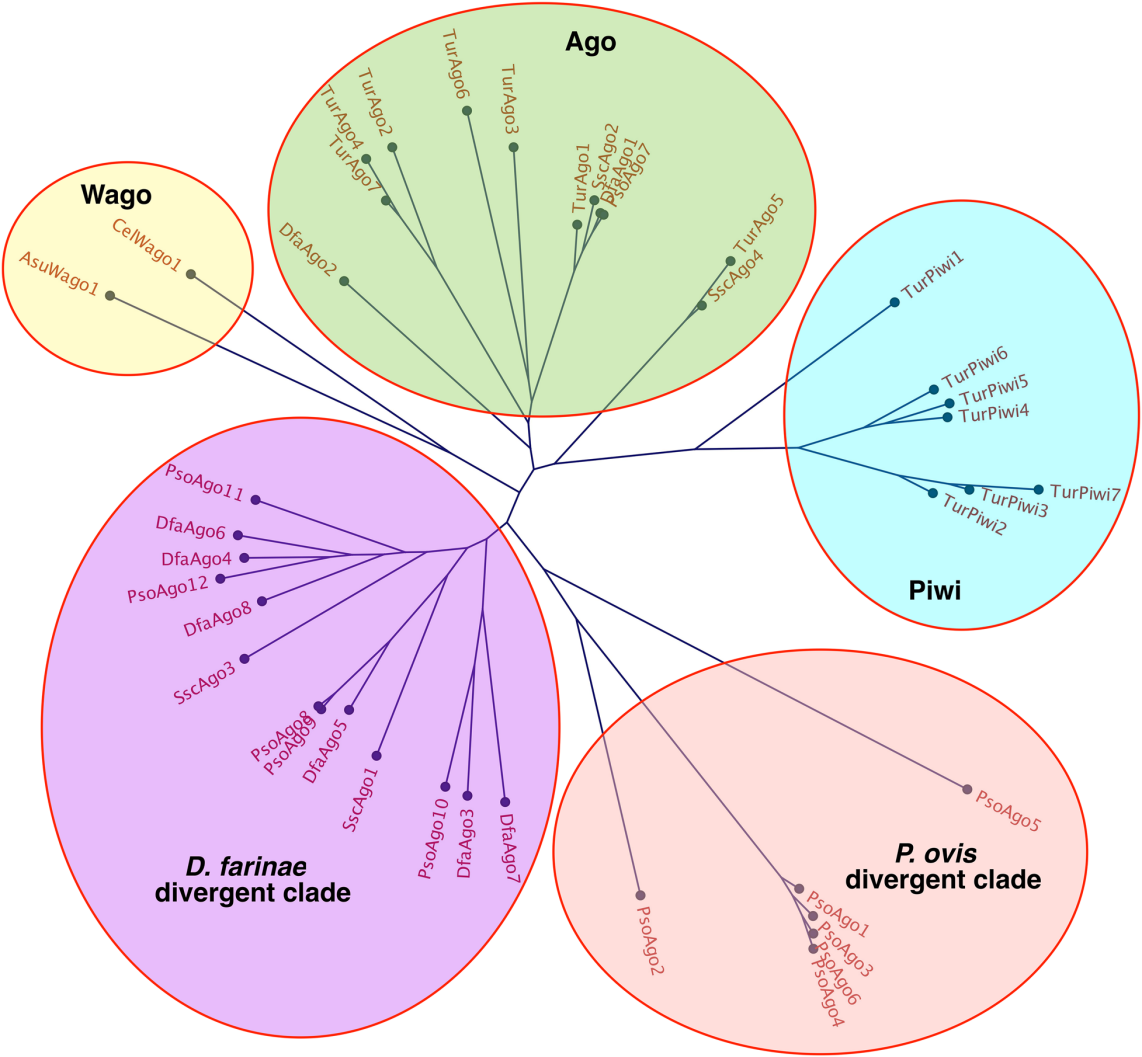
**Phylogentic sequence analysis of** ***P. ovis*** **argonaute proteins reveals loss of piwi-clade argonautes within** ***Psoroptes ovis***. *S. scabiei* Ago proteins: SscAgo1 (KPM04840.1); SscAgo2 (KPM09009.1); SscAgo3 (KPM04151.1); SscAgo4 (KPM09008.1). *T. urticae* Ago proteins: TurAgo1 (tetur20g02910); TurAgo2 (tetur09g03140); TurAgo3 (tetur09g00620); TurAgo4 (tetur02g10560); TurAgo5 (tetur02g10580); TurAgo6 (tetur04g01190); TurAgo7 (tetur02g10570). *T. urticae* Piwi proteins: TurPiwi1 (tetur06g03300); TurPiwi2 (tetur28g00450); TurPiwi3 (tetur28g00340); TurPiwi4 (tetur06g05570); TurPiwi5 (tetur06g05580); TurPiwi6 (tetur06g05600); TurPiwi7 (tetur17g03380). *D. farinae* Ago proteins: DfaAgo1 (AUI38415.1); DfaAgo2 (AUI38416.1); DfaAgo3 (AUI38417.1); DfaAgo4 (AUI38418.1); DfaAgo5 (AUI38419.1); DfaAgo6 (AUI38420.1); DfaAgo7 (AUI38421.1); DfaAgo8 (AUI38422.1). *P. ovis* Ago proteins: PsoAgo1 (psovi14g07720); PsoAgo2 (psovi14g07690); PsoAgo3 (psovi14g07700); PsoAgo4 (psovi14g07730); PsoAgo5 (psovi63g00040); PsoAgo6 (psovi14g07710); PsoAgo7 (psovi295g00210); PsoAgo8 (psovi280g02500); PsoAgo9 (psovi280g02490); PsoAgo10 (psovi26g02770); PsoAgo11 (psovi294g02030); PsoAgo12 (psovi302g00450). *C. elegans* Wago-1 (CelWago1: Q21770-1); *Ascaris suum* Wago-1 (AsuWago1: F1L7U8-1).

Homologous sequences to the major RNAi pathway components (identified from *T. urticae*, *C. elegans* and *D. melanogaster*) were identified in the *P. ovis* genome, including sequences encoding genes involved in RNA induced silencing complex (RISC) formation, small RNA biosynthesis and secondary amplification of small RNAs (Table 1). Notably absent were the dsRNA uptake and spreading components sid-1, sid-2, rsd-2 and rsd-6, which are known to mediate the uptake of extracellular dsRNA and also the intracellular spreading of dsRNA [26]. In particular, sid-1 has been shown in a number of species, including *C. elegans* and *Bombyx mori*, to play a critical role in the demonstration of robust RNAi by mediating cellular uptake of dsRNA in somatic and germ-line cells [27–29]. Piwi-clade argonautes were also absent from the *P. ovis* genome, an observation recently highlighted in the context of the relatively closely related Psoroptidae, the American house dust mite *Dermatophagoides farinae* [30] (Figure 1). Furthermore, as previously reported for *D. farinae*, we also identified a similar divergent clade of ago proteins in *P. ovis*, with five members (PsoAgo8-PsoAgo12, Figure 1). These divergent ago proteins share an unusual DEDD catalytic motif, compared to the more common DEDH slicer motif found in metazoan Ago and Piwi proteins [31]. Orthologs with the same DEDD motif have been identified in *S. scabiei*, *C. elegans* and the social spider, *Stegodyphus mimosarum* [32–34]. In addition, a further divergent clade of potential *P. ovis*-specific ago proteins with six members was identified (PsoAgo1–PsoAgo6, Figure 1) of which, PsoAgo 1, 3, 4 and 6 contain an atypical slicer motif (S[E/K]HE).

**Table 1:** **RNAi pathway components identified in the** ***Psoroptes ovis*** **genome**

Gene family (Organism)	Accession ID^a^	*P. ovis* gene ID^b^	Gene number	Top blast hit E-value
Small RNA biosynthesis				
Dicer (*T. urticae*)	tetur19g00520; tetur07g00990	psovi14g04930; psovi280g04020; psovi298g00280 psovi14g04950; psovi280g04030; psovi14g04940	6	7e−117
Drosha (*T. urticae*)	tetur12g00910	psovi45g00290	1	0.0
drh-1 (*C. elegans*)	F15B10.2	psovi288g01850; psovi14g00590; psovi288g01290	3	2e−11
Exportin-5 (*T. urticae*)	tetur02g00520; tetur02g00500	psovi17g08260	1	2e−05
Loquacious (*T. urticae*)	tetur13g00430; tetur13g00410	psovi14g10890; psovi292g01850; psovi283g06140	3	1e−31
Pasha (*T. urticae*)	tetur36g00220; tetur36g00250	psovi05g03810	1	1e−161
xpo-1 (*C. elegans*)	ZK742.1	psovi36g04430	1	0.0
xpo-2 (*T. urticae*)	tetur18g01350	psovi09g00130; psovi52g00090	2	0.0
RNA induced silencing complex (RISC) components				
ain-2 (*C. elegans*)	B0041.2	psovi 298g00740	1	4e−04
C3P0 (*Tribolum castaneum*)	ABX72055	N/A	N/A	N/A
R2d2 (*D. melanogaster*)	NP_001285720.1	N/A	N/A	N/A
tsn-1 (*C. elegans*)	F10G7.2	psovi90g00040	1	4e−123
vig-1 (*T. urticae*)	tetur22g01310	psovi301g01900	1	9e−22
RNAi inhibitors				
adr-2 (*C. elegans*)	T20H4.4	psovi280g06440	1	9e−24
eri-1 (*C. elegans*)	T07A9.5	psovi17g06910	1	6e−28
eri-6/7 (*C. elegans*)	C41D11.1	psovi286g03350; psovi66g00290; psovi286g02110; psovi05g05100	4	9e−32
xrn-1 (*C. elegans*)	Y39G8C.1	psovi283g01010	1	0.0
xrn-2 (*T. urticae*)	tetur25g00400	psovi17g08800	1	0.0
dsRNA uptake and spreading				
GW182 (*T. urticae*)	tetur05g07970; tetur09g00260	psovi294g01730; psovi17g04680	2	1e−18
rsd-3 (*C. elegans*)	C34E11.1	psovi22g08140; psovi20g00980	2	4e−41
Secondary amplification				
RdRP (*T. urticae*)	tetur02g08750; tetur02g08760; tetur02g08780; tetur02g08810 tetur02g08820	psovi20g00680; psovi69g00210; psovi14g08400	3	1e−61
Nuclear effectors				
cid-1 (*C. elegans*)		psovi280g05740; psovi22g06910; psovi17g01850 psovi40g00030; psovi08g01430; psovi38g00730 psovi35g01570; psovi33g01410; psovi283g04640 psovi26g00430; psovi08g01710	11	3e−09
gfl-1 (*C. elegans*)	M04B2.3	psovi14g08490; psovi283g02390	2	2e−41
mes-2 (*C. elegans*)	R06A4.7	psovi17g07450; psovi283g06100; psovi301g02970 psovi05g04620; psovi05g01520; psovi58g00680	6	3e−56
mes-6 (*C. elegans*)	C09G4.5	psovi280g00380; psovi14g10140	2	7e−12
mut-2 (*C. elegans*)	K04F10.6	psovi44g00310	1	2e−10
mut-16 (*C. elegans*)	B0379.3	psovi33g00580	1	5e−04
rha-1 (*C. elegans*)	T07D4.3	psovi45g00690; psovi14g03610; psovi22g06890 psovi26g00980; psovi17g03400; psovi22g00930 psovi43g00020; psovi14g10090; psovi298g00430	9	2e−128
zfp-1 (*C. elegans*)	F54F2.2	psovi26g03300; psovi17g07470; psovi09g00270; psovi294g01700; psovi09g02230	5	4e−54
RISC—argonaute/piwi proteins				
Argonaute (*T. urticae*; *D. farinae*)	tetur20g02910; tetur09g03140 tetur09g00620; tetur02g10560 tetur02g10580; tetur04g01190 tetur02g10570; KY794591; KY794592	psovi295g00210	1	0.0
*D. farinae* Divergent Ago Clade	KY794593; KY794594; KY794598; KY794596; KY794597; KY794598	psovi280g02500; psovi280g02490; psovi26g02770; psovi294g02030; psovi302g00450	5	0.0
*P. ovis* Divergent Ago Clade	N/A	psovi14g07720; psovi14g07690; psovi14g07730; psovi14g07700; psovi14g07710; psovi63g00040	6	N/A
Piwi (*T. urticae*)	tetur06g03300 tetur28g00450; tetur28g00340 tetur06g05570; tetur06g05580 tetur06g05600; tetur17g03380	N/A	N/A	N/A
nrde-1 (*T. urticae*)	tetur21g02920	psovi17g01230	1	5e−71

^a^*Tetranychus urticae* accession numbers from Grbic et al. [47] (in format tetur00g00000).

^b^*Psoroptes ovis* accession numbers (in format psovi00g00000).

Finally, in addition to the RNAi pathway-specific genes, components of the microRNA (miRNA) pathway were detected, including argonaute-1, drosha (drsh) and partner of drosha (DGCR8), which is involved in primary-miRNA (pri-miRNA) processing [35]. RNAi pathway components including dcr-1 and xpo-1 are also involved in the miRNA pathway, processing pre-miRNA into mature miRNA [36–38] and exporting nuclear pri-miRNAs respectively [39]. Tudor staphylococcal nuclease (tsn-1) and exoribonuclease 2 (xrn-2) were also identified in *P. ovis* (Table 1). Tsn-2 has been shown to impact on let-7 miRNA function in addition to a known involvement in RISC complexes in *Drosophila melanogaster* and *C. elegans* [40, 41] and xrn-2 is likely to be involved in miRNA degradation [42].

### Validation of non-invasive RNAi methodology in *P. ovis* mites

**Figure 2:**
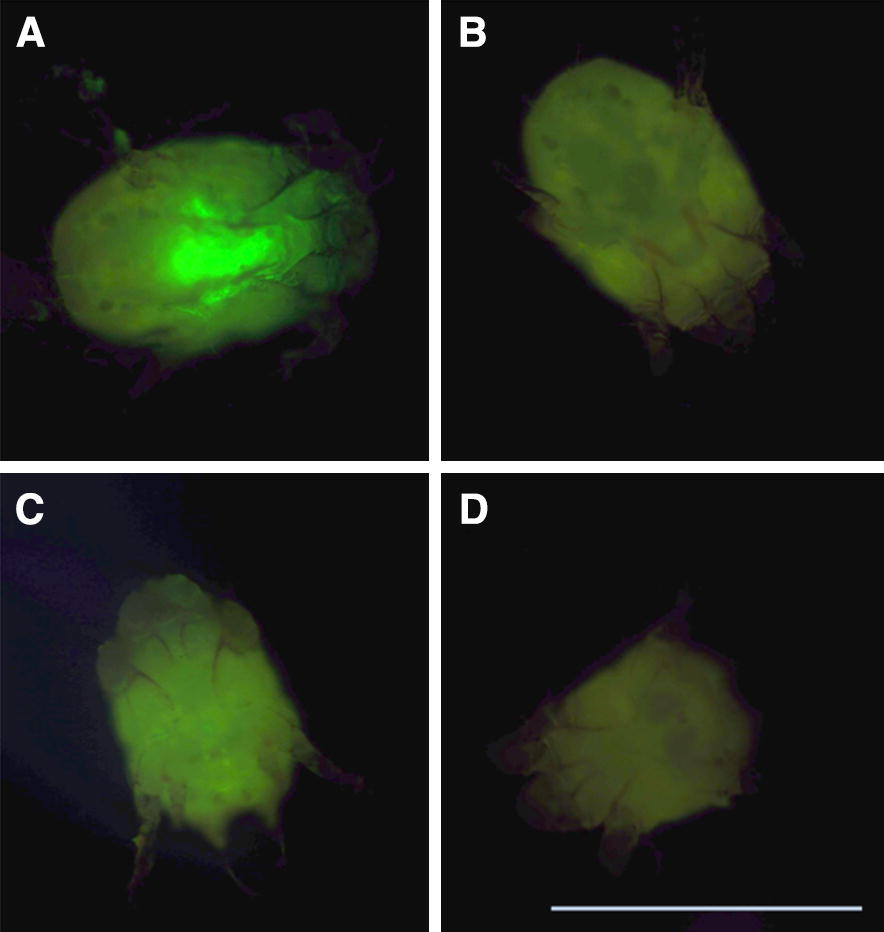
**Fluoro-siRNA uptake in female and male** ***Psoroptes ovis*** **mites.** Adult female and male *P. ovis* mites were immersed overnight in fluoro-siRNA solution (**A**, **C**, respectively) or 0.9% w/v NaCl solution (**B**, **D**, respectively). Mites were viewed by CFL inverted fluorescent microscope and images captured using a Nikon digital SLR camera. Scale bar: 500 µm.

The results of the fluoro-siRNA uptake experiment demonstrated uptake of siRNA in adult female *P. ovis* mites (Figure 2A). The fluorescence in the fluoro-siRNA immersed mites was most intense at the anterior end of the hysterosoma where the foregut is located (Figure 2A). Limited auto-fluorescence was also detected in the 0.9% w/v NaCl solution-immersed control mites, although this was substantially less intense compared to the fluoro-siRNA immersed mites and more generalised across the idiosoma in its entirety, with no localisation to the gut structure (Figure 2B). Uptake was visible as increased fluorescence compared to controls from 1 h, increasing until 9 h, after which there was no discernible difference in fluorescence between 9 and 24 h (data not shown). Uptake of siRNA was not achieved by immersion of *P. ovis* eggs in fluoro-siRNA as there was no difference observed between eggs immersed in fluoro-siRNA (0.05 µg/µL) and control eggs immersed in 0.9% w/v NaCl solution (not shown). In male mites immersed in fluoro-siRNA, fluorescence was also most intense at the anterior-most end of the hysterosoma where the foregut/anterior midgut is located (Figure 2C) with limited auto-fluorescence detected in control male mites (Figure 2D).

### Gene silencing by RNAi in *P. ovis* mites

**Figure 3:**
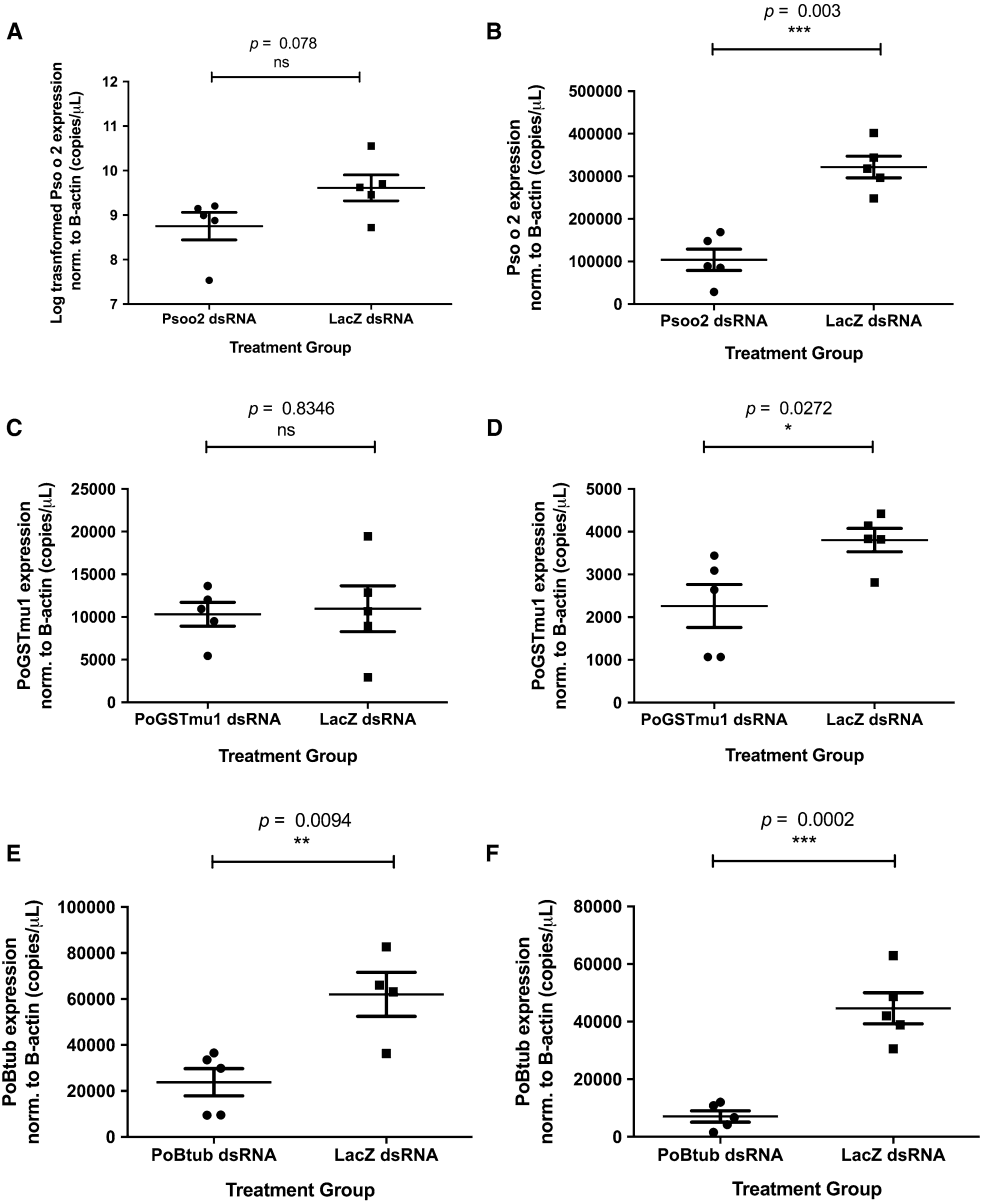
**Gene silencing in** ***Psoroptes ovis*** **by RNAi.** Mean expression levels of transcripts encoding Pso o 2, *Po*GST-mu1 and *Po*βtub normalised to *Po*βactin (copies/µL) determined by qPCR compared to control mites immersed in dsRNA encoding lacZ are shown for adult male mites immersed overnight at 4 °C in dsRNA representing Pso o 2 (**A**), *Po*GST-mu1 (**C**) and *Po*βtub (**E**), or immersed in dsRNA representing Pso o 2 (**B**), *Po*GST-mu1 (**D**) and *Po*βtub (**F**) overnight at 4 °C followed by 48 h in a humidity incubator (25 °C, 75% RH). *n* = 5 for each treatment group with the exception of the lacZ control group in the *Po*βtub overnight incubation only experiment where *n* = 4, error bars indicate mean ± SEM.

Immersion of adult male *P. ovis* mites for 24 h in dsRNA representing Pso o 2, *Po*GST-mu1 and *Po*βtub at 4 °C followed by removal of the dsRNA and a subsequent 48 h period at 25 °C, 75% relative humidity resulted in statistically significantly reduced mRNA transcript levels (*p* = 0.003, *p* = 0.0272, *p* = 0.0002 respectively), compared with mites immersed in control lacZ dsRNA (Figure 3). Mean reductions in transcript levels relative to the lacZ control were 67.7%, 40.5% and 84.2% respectively for Pso o 2, *Po*GST-mu1 and *Po*βtub. Immersion in gene-specific dsRNA at 4 °C for 24 h alone was not sufficient to produce significant decreases in transcripts representing Pso o 2 or *Po*GST-mu1, however, immersion at 4 °C for 24 h alone was sufficient to achieve a significant reduction in Poβtub expression compared to the lacZ control, averaging 61.6% (*p* = 0.0094). Log transformation of raw data was required for the 24 h immersion of Pso o 2 versus lacZ dsRNA (Figure 3A) due to unequal variance between treatment groups. No adverse phenotypic effects following gene knockdown were observed in any of the experiments.

## Discussion

Here we have demonstrated the presence of an RNAi pathway in *P. ovis* through the bioinformatic analysis of the recently published *P. ovis* genome. Furthermore, we have validated this as an active pathway using a non-invasive dsRNA delivery approach, demonstrating significant gene silencing of three mite genes.

Homologues of several known RNAi pathway components were detected in the genome (Table 1) and the demonstration of gene silencing by RNAi shown here validated the functionality of the pathway in *P. ovis*. As discussed in the review by Marr et al. [43] the presence of RNAi pathway components is a promising indicator for the successful demonstration of RNAi. The components identified by tBlastx analysis of the *P. ovis* genome indicate the presence of effectors of the basic machinery required for processing dsRNA or small RNA biosynthesis amongst other key aspects of the RNAi pathway. For example, homologous sequences to dicer, argonaute and exportin strongly suggests these genes are present in *P. ovis,* implying it has the machinery required to process dsRNA into siRNAs. Components of the RNA induced silencing complex (RISC) were identified, suggesting that *P. ovis* is capable of incorporating those siRNAs generated by the Dicer processing of dsRNA. Furthermore, homologous sequence to RNA-directed RNA Polymerases (RdRP) Ego-1 and Rrf-1 were detected. These are considered essential for the amplification of dsRNA-induced RNAi in *C. elegans* and the thale cress plant *Arabidopsis thaliana* [44, 45] although not essential for systemic RNAi in the red flour beetle, *Tribolium castaneum* [46]. Orthologues of RdRPs have been detected in *T. urticae* [47] *Ixodes scapularis* and *Rhipicephalus microplus* indicating that some members of the Acari do express RdRPs and it appears that *P. ovis* might also express RdRPs, with *T. urticae* RdRPs blast hits at the amino acid level being highly comparable to the *P. ovis* RdRP matches (Table 1). This may explain the relatively rapid onset of silencing in *P. ovis*, with at least one of the targets tested here, with significant gene silencing demonstrable within 24 h, suggesting that spreading or systemic RNAi may occur. Although no research exists on the viral infections challenging *P. ovis* mites, research in the model fruit fly, *Drosophila melanogaster* has demonstrated the necessity of dsRNA uptake pathways in order to mount protective immune responses to viral challenge; this may provide an explanation as to why many elements of the RNAi pathway were detected in the *P. ovis* genome [48].

Although there were some notable absences, for example sid-1 within the category of dsRNA uptake and spreading, rsd-3 was detected in the *P. ovis* genome (Table 1) and may play a role in systemic RNAi through intracellular vesicular transport of ds/siRNA suggesting *P. ovis* might be capable of systemic RNAi [26]. A recent study highlighted the absence of piwi clade argonautes and their associated piRNAs within the American house dust mite *D. farinae* [30]. A homology-based search of the *P. ovis* and *S. scabiei* genomes indicated that this loss may be at least partially conserved amongst Psoroptidae mites (Figure 1). Furthermore, this loss may account for the expansion of species-specific argonautes revealed through phylogenetic evaluation of available argonaute sequences within *P. ovis*, *D. farinae* and *S. scabiei* when compared to *T. urticae* (Figure 1).

Uptake of dsRNA by immersion was demonstrated in both male and female *P. ovis* (Figure 2). Based on the absence of RNA uptake in eggs, we decided to avoid the use of gravid females for gene silencing experiments, as their eggs could act as an RNAi-inaccessible reservoir for transcripts. Uptake of dsRNA was most probably via ingestion on the basis that *P. ovis* is an astigmatid mite; thereby having no stigmata and thus relying on ingestion to maintain water homeostasis. Topical application of dsRNA solution to the cuticle of the mesostigmatid mite, *Varroa destructor* did not result in gene silencing, suggesting that the cuticle is impermeable to dsRNA solution and further supporting the theory of ingestion resulting in uptake of dsRNA. Lipofectamine reagent is commonly used to achieve RNAi in parasitic nematodes such as *Haemonchus contortus* where it has been demonstrated to improve gene silencing, possibly by increasing uptake of dsRNA within the gut cells due to the positive charge of the liposomes encapsulating the negatively charged dsRNA [49]. The use of lipofectamine did not result in enhancement of the silencing effect in *P. ovis* across the parameters assessed (data not shown). A similar lack of enhancement was demonstrated recently in *D. pteronyssinus* [20] although whether this applies to the two step incubator protocol in the present study and/or to further targets remains to be determined. Similarly, the use of phagostimulants was considered during the development of the protocol however existing experimental data [50] indicates common phagostimulants such as adenosine triphosphate (ATP) did not enhance *P. ovis* feeding, although their effect in RNAi immersion experiments has yet to be determined and could form the basis of future optimisation experiments. The benefits of phagostimulants have been investigated in the context of tick feeding in *Ornithodoros tholozani*, with maximal intake of food achieved with the use of ATP, reduced nicotinamide adenine dinucleotide (NADH) or (*S*)-2-aminopentanedioic acid (l-glutatmate) [51]. Glutathione was also shown to moderately increase feeding in *O. tholozani* but the converse, reduced feeding, was seen in *O. moubata* [52].

There were target-specific variations in the degree of silencing amongst the three transcripts targeted here and also in the time at which silencing appears to take effect, or at least become significantly reduced compared to the control. These differences may pertain to localisation of the target mRNA transcripts and/or their relative level of expression and rate of transcription. There were no detrimental phenotypic consequences evident following significant gene silencing in any of the three genes targeted. This result was not entirely unexpected, given that observations were limited to the experimental period (maximum 72 h). Furthermore, the genes targeted (with the possible exception of *Po*βtub) had no predicted lethal outcome following silencing given the roles they play within the mite in vivo: no specific functional role has yet been assigned, or demonstrated, for Pso o 2, but it is hypothesised to play a key role in establishing infestation by acting as a functional mimic of the toll-like receptor-4 (TLR4) accessory protein, MD-2; as demonstrated for the *D. pteronyssinus* orthologue, Der p 2 [53]. The phenotypic implications of Pso o 2 gene silencing are therefore likely only to be seen if the mites were returned to the host following successful RNAi. The results relating to *Po*GST-mu1 in *P. ovis* are consistent with those detailed previously in *D. pteronyssinus*, *S. scabiei* and *V. destructor*, where no detrimental phenotypic effects were evident following significant gene silencing of *Dp*/*Vd*GST-mu1 by RNAi [19, 20, 23]. GSTs are widely implicated in the development of drug resistance and act by conjugating glutathione to toxic endo- and xeno-biotics, rendering them more hydrophilic and thus more amenable to excretion [54]. The use of abiotic stressors that might result in an increased need for GST detoxification activity in RNAi experiments were discussed in Marr et al. [20] and are also relevant for *P. ovis*. Similar approaches have been adopted for other arthropod pests such as the cotton aphid, *Aphis gossypii*, where RNAi gene silencing of carboxylesterases resulted in reduced resistance to organophosphate-based insecticides [55]. GSTs are a multi-class family of enzymes and several classes have been identified within *P. ovis*, including delta, mu, alpha and epsilon class GSTs [56]. It is possible that there could be functional redundancy among the *P. ovis* GSTs, thus avoiding any potential detrimental phenotypic outcome following the successful silencing of one class member.

The use of RNAi in vitro for the identification of promising vaccine candidates or other novel control mechanisms has both potential and limitations (as discussed [43]). For example, here we demonstrated that silencing of the genes encoding Pso o 2 and *Po*GST-mu1 has no immediate phenotypic effect but the proteins encoded by both of these genes are components of a promising recombinant cocktail vaccine against *P. ovis* [57]. The future use of RNAi in *P. ovis* to identify promising vaccine candidates and/or other novel interventions may require in vitro silencing of genes followed by subsequent challenge infestation of host animals to determine the presence of any detrimental phenotypic effects, particularly when genes involved in host:parasite interactions are the targets. Such in vivo trials would require information pertaining to the longevity of gene silencing for each gene assessed and should focus on those genes that are likely to play a role in the initial onset of infestation such as the proteolytic allergen, Pso o 1 and the putative MD-2 mimic, Pso o 2. In these cases, the ability of mites to establish infestation following RNAi gene silencing could be assessed to further characterise a candidate gene’s therapeutic potential.

This is the first demonstration of gene silencing by RNAi in *P. ovis* and paves the way for further RNAi studies in this important ectoparasite. Scaling up and adaptation of the RNAi protocol demonstrated here in order to perform genome-wide, in vitro and in vivo screens is an exciting possibility for future development. Genome-wide RNAi screens have demonstrated great potential in highlighting essential genes [58, 59] and also highlighting the relationships with organisms symbionts [60]. This approach could prove to be highly beneficial for discovering novel candidates for control of this important ectoparasitic mite.

## References

[1] Burr E (1984). *Psoroptes ovis* isolated from a giraffe (Giraffa camelopardalis) in Massai Mara Game Reserve Kenya. Kenyan Vet.

[2] El-Khodery SA, Osman SA, Ishii M, Al-Gaabary MH (2010). Risk factors of infestation by *Psoroptes* spp. mites in buffalo (Bubalus bubalis) at smallholder farms in the Nile Delta region, Egypt. Trop Anim Health Prod.

[3] Karbowiak G, Demiaszkiewicz AW, Pyziel AM, Wita I, Moskwa B, Werszko J, Bien J, Gozdzik K, Lachowicz J, Cabaj W (2014). The parasitic fauna of the European bison (Bison bonasus) (Linnaeus, 1758) and their impact on the conservation. Part 1. The summarising list of parasites noted. Acta Parasitol.

[4] Gabaj MM, Beesley WN, Awan MA (1992). A survey of mites on farm animals in Libya. Ann Trop Med Parasitol.

[5] Lange RE, Sandoval AV, Meleney WP (1980). Psoroptic scabies in bignorn sheep (Ovis canadensis mexicana) in New Mexico. J Wildl Dis.

[6] Zahler M, Hendrikx WM, Essig A, Rinder H, Gothe R (2000). Species of the genus psoroptes (Acari: Psoroptidae): a taxonomic consideration. Exp Appl Acarol.

[7] Coles GC (1998). Drug-resistant parasites of sheep: an emerging problem in Britain?. Parasitol Today.

[8] McNair CM, Nisbet AJ, Billingsley PF, Knox DP (2009). Molecular characterization, expression and localization of a peroxiredoxin from the sheep scab mite, *Psoroptes ovis*. Parasitology.

[9] Kirkwood AC (1980). Effect of *Psoroptes ovis* on the weight of sheep. Vet Rec.

[10] Kirkwood AC (1985). Some observations on the biology and control of the sheep scab mite *Psoroptes ovis* (Hering) in Britain. Vet Parasitol.

[11] Corke MJ, Broom DM (1999). The behaviour of sheep with sheep scab, *Psoroptes ovis* infestation. Vet Parasitol.

[12] Sargison ND (1995). Differential diagnosis and treatment of sheep scab. Practice.

[13] Sargison ND, Scott PR, Penny CD, Pirie RS (1995). Effect of an outbreak of sheep scab (*Psoroptes Ovis* infestation) during mid-pregnancy on ewe body condition and lamb birth-weight. Vet Rec.

[14] Bygrave AC, Bates PG, Daniel NJ (1993). Epileptiform seizure in ewes associated with sheep scab mite infestation. Vet Rec.

[15] Buchanan D, Pilkington A, Sewell C, Tannahill SN, Kidd MW, Cherrie B, Hurley JF (2001). Estimation of cumulative exposure to organophosphate sheep dips in a study of chronic neurological health effects among United Kingdom sheep dippers. Occup Environ Med.

[16] Pilkington A, Buchanan D, Jamal GA, Gillham R, Hansen S, Kidd M, Hurley JF, Soutar CA (2001). An epidemiological study of the relations between exposure to organophosphate pesticides and indices of chronic peripheral neuropathy and neuropsychological abnormalities in sheep farmers and dippers. Occup Environ Med.

[17] Doherty E, Burgess S, Mitchell S, Wall R (2018). First evidence of resistance to macrocyclic lactones in *Psoroptes ovis* sheep scab mites in the UK. Vet Rec.

[18] Burgess STG, Bartley K, Marr EJ, Wright HW, Weaver RJ, Prickett JC, Hughes M, Haldenby S, Le Thi P, Rombauts S, Van Leeuwen T, Van de Peer Y, Nisbet AJ (2018). Draft genome assembly of the sheep scab mite, *Psoroptes ovis*. Genome Announc.

[19] Fernando DD, Marr EJ, Zakrzewski M, Reynolds SL, Burgess STG, Fischer K (2017). Gene silencing by RNA interference in *Sarcoptes scabiei:* a molecular tool to identify novel therapeutic targets. Parasit Vectors.

[20] Marr EJ, Sargison ND, Nisbet AJ, Burgess ST (2015). Gene silencing by RNA interference in the house dust mite, *Dermatophagoides pteronyssinus*. Mol Cell Probes.

[21] Maule AG, McVeigh P, Dalzell JJ, Atkinson L, Mousley A, Marks NJ (2011). An eye on RNAi in nematode parasites. Trends Parasitol.

[22] Altschul SF, Gish W, Miller W, Myers EW, Lipman DJ (1990). Basic local alignment search tool. J Mol Biol.

[23] Campbell EM, Budge GE, Bowman AS (2010). Gene-knockdown in the honey bee mite Varroa destructor by a non-invasive approach: studies on a glutathione *S*-transferase. Parasit Vectors.

[24] Rozen S, Skaletsky H (2000). Primer3 on the WWW for general users and for biologist programmers. Methods Mol Biol.

[25] Burgess STG, Downing A, Watkins CA, Marr EJ, Nisbet AJ, Kenyon F, McNair C, Huntley JF (2012). Development of a cDNA microarray for the measurement of gene expression in the sheep scab mite *Psoroptes ovis*. Parasit Vectors.

[26] Tijsterman M, May RC, Simmer F, Okihara KL, Plasterk RHA (2004). Genes required for systemic RNA interference in *Caenorhabditis elegans*. Curr Biol.

[27] Winston WM, Molodowitch C, Hunter CP (2002). Systemic RNAi in *C. elegans* requires the putative transmembrane protein SID-1. Science.

[28] Feinberg EH, Hunter CP (2003). Transport of dsRNA into cells by the transmembrane protein SID-1. Science.

[29] Kobayashi I, Tsukioka H, Komoto N, Uchino K, Sezutsu H, Tamura T, Kusakabe T, Tomita S (2012). SID-1 protein of *Caenorhabditis elegans* mediates uptake of dsRNA into Bombyx cells. Insect Biochem Mol Biol.

[30] Mondal M, Klimov P, Flynt AS (2018). Rewired RNAi-mediated genome surveillance in house dust mites. PLoS Genet.

[31] Parker JS, Roe SM, Barford D (2004). Crystal structure of a PIWI protein suggests mechanisms for siRNA recognition and slicer activity. EMBO J.

[32] Rider SD, Morgan MS, Arlian LG (2015). Draft genome of the scabies mite. Parasit Vectors.

[33] Sanggaard KW, Bechsgaard JS, Fang X, Duan J, Dyrlund TF, Gupta V, Jiang X, Cheng L, Fan D, Feng Y, Han L, Huang Z, Wu Z, Liao L, Settepani V, Thogersen IB, Vanthournout B, Wang T, Zhu Y, Funch P, Enghild JJ, Schauser L, Andersen SU, Villesen P, Schierup MH, Bilde T, Wang J (2014). Spider genomes provide insight into composition and evolution of venom and silk. Nat Commun.

[34] Faehnle CR, Joshua-Tor L (2007). Argonautes confront new small RNAs. Curr Opin Chem Biol.

[35] Han J, Lee Y, Yeom KH, Kim YK, Jin H, Kim VN (2004). The Drosha-DGCR8 complex in primary microRNA processing. Genes Dev.

[36] Grishok A, Pasquinelli AE, Conte D, Li N, Parrish S, Ha I, Baillie DL, Fire A, Ruvkun G, Mello CC (2001). Genes and mechanisms related to RNA interference regulate expression of the small temporal RNAs that control *C. elegans* developmental timing. Cell.

[37] Ketting RF, Fischer SE, Bernstein E, Sijen T, Hannon GJ, Plasterk RH (2001). Dicer functions in RNA interference and in synthesis of small RNA involved in developmental timing in *C. elegans*. Genes Dev.

[38] Hutvagner G, McLachlan J, Pasquinelli AE, Balint E, Tuschl T, Zamore PD (2001). A cellular function for the RNA-interference enzyme Dicer in the maturation of the let-7 small temporal RNA. Science.

[39] Bussing I, Yang JS, Lai EC, Grosshans H (2010). The nuclear export receptor XPO-1 supports primary miRNA processing in *C. elegans* and *Drosophila*. EMBO J.

[40] Caudy AA, Ketting RF, Hammond SM, Denli AM, Bathoorn AM, Tops BB, Silva JM, Myers MM, Hannon GJ, Plasterk RH (2003). A micrococcal nuclease homologue in RNAi effector complexes. Nature.

[41] Murchison EP, Hannon GJ (2004). miRNAs on the move: miRNA biogenesis and the RNAi machinery. Curr Opin Cell Biol.

[42] Gy I, Gasciolli V, Lauressergues D, Morel JB, Gombert J, Proux F, Proux C, Vaucheret H, Mallory AC (2007). Arabidopsis FIERY1, XRN2, and XRN3 are endogenous RNA silencing suppressors. Plant Cell.

[43] Marr EJ, Sargison ND, Nisbet AJ, Burgess STG (2014). RNA interference for the identification of ectoparasite vaccine candidates. Parasite Immunol.

[44] Sijen T, Fleenor J, Simmer F, Thijssen KL, Parrish S, Timmons L, Plasterk RH, Fire A (2001). On the role of RNA amplification in dsRNA-triggered gene silencing. Cell.

[45] Smardon A, Spoerke JM, Stacey SC, Klein ME, Mackin N, Maine EM (2000). EGO-1 is related to RNA-directed RNA polymerase and functions in germ-line development and RNA interference in *C. elegans*. Curr Biol.

[46] Tomoyasu Y, Miller SC, Tomita S, Schoppmeier M, Grossmann D, Bucher G (2008). Exploring systemic RNA interference in insects: a genome-wide survey for RNAi genes in *Tribolium*. Genome Biol.

[47] Grbic M, Van Leeuwen T, Clark RM, Rombauts S, Rouze P, Grbic V, Osborne EJ, Dermauw W, Ngoc PC, Ortego F, Hernandez-Crespo P, Diaz I, Martinez M, Navajas M, Sucena E, Magalhaes S, Nagy L, Pace RM, Djuranovic S, Smagghe G, Iga M, Christiaens O, Veenstra JA, Ewer J, Villalobos RM, Hutter JL, Hudson SD, Velez M, Yi SV, Zeng J (2011). The genome of *Tetranychus urticae* reveals herbivorous pest adaptations. Nature.

[48] Saleh MC, Tassetto M, van Rij RP, Goic B, Gausson V, Berry B, Jacquier C, Antoniewski C, Andino R (2009). Antiviral immunity in *Drosophila* requires systemic RNA interference spread. Nature.

[49] Kotze AC, Bagnall NH (2006). RNA interference in *Haemonchus contortus*: suppression of beta-tubulin gene expression in L3, L4 and adult worms in vitro. Mol Biochem Parasitol.

[50] Deloach JR (1984). In vitro feeding of *Psoroptes ovis* (Acari:Psoroptidae). Vet Parasitol.

[51] Galun R, Kindler SH (1968). Chemical basis of feeding in the tick *Ornithodoros tholozani*. J Insect Physiol.

[52] Ben-Yakir D, Galun R (1993). Comparative study of two argasid tick species: feeding response to phagostimulants. Isr J Zool.

[53] Trompette A, Divanovic S, Visintin A, Blanchard C, Hegde RS, Madan R, Thorne PS, Wills-Karp M, Gioannini TL, Weiss JP, Karp CL (2009). Allergenicity resulting from functional mimicry of a Toll-like receptor complex protein. Nature.

[54] Salinas AE, Wong MG (1999). Glutathione S-transferases—a review. Curr Med Chem.

[55] Gong YH, Yu XR, Shang QL, Shi XY, Gao XW (2014). Oral delivery mediated RNA interference of a carboxylesterase gene results in reduced resistance to organophosphorus insecticides in the cotton Aphid, *Aphis gossypii* Glover. PLoS One.

[56] Burgess ST, Nisbet AJ, Kenyon F, Huntley JF (2011). Generation, analysis and functional annotation of expressed sequence tags from the ectoparasitic mite *Psoroptes ovis*. Parasit Vectors.

[57] Burgess STG, Nunn F, Nath M, Frew D, Wells B, Marr EJ, Huntley JF, McNeilly TN, Nisbet AJ (2016). A recombinant subunit vaccine for the control of ovine psoroptic mange (sheep scab). Vet Res.

[58] Kamath RS, Ahringer J (2003). Genome-wide RNAi screening in *Caenorhabditis elegans*. Methods.

[59] Kamath RS, Fraser AG, Dong Y, Poulin G, Durbin R, Gotta M, Kanapin A, Le Bot N, Moreno S, Sohrmann M, Welchman DP, Zipperlen P, Ahringer J (2003). Systematic functional analysis of the *Caenorhabditis elegans* genome using RNAi. Nature.

[60] Taracena ML, Oliveira PL, Almendares O, Umana C, Lowenberger C, Dotson EM, Paiva-Silva GO, Pennington PM (2015). Genetically modifying the insect gut microbiota to control chagas disease vectors through systemic RNAi. PLoS Negl Trop Dis.

